# Thermostable Bacterial Collagenolytic Proteases: A Review

**DOI:** 10.4014/jmb.2404.04051

**Published:** 2024-06-17

**Authors:** Kui Zhang, Yapeng Han

**Affiliations:** 1 College of Life Sciences and Technology, Longdong University, Qingyang 745000, P.R. China; 2 Gansu Key Laboratory of Protection and Utilization for Biological Resources and Ecological Restoration, Qingyang 745000, P.R. China

**Keywords:** Collagen, collagenolytic protease, collagenase, thermostable subtilisin-like serine protease, TSS

## Abstract

Collagenolytic proteases are widely used in the food, medical, pharmaceutical, cosmetic, and textile industries. Mesophilic collagenases exhibit collagenolytic activity under physiological conditions, but have limitations in efficiently degrading collagen-rich wastes, such as collagen from fish scales, at high temperatures due to their poor thermostability. Bacterial collagenolytic proteases are members of various proteinase families, including the bacterial collagenolytic metalloproteinase M9 and the bacterial collagenolytic serine proteinase families S1, S8, and S53. Notably, the C-terminal domains of collagenolytic proteases, such as the pre-peptidase C-terminal domain, the polycystic kidney disease-like domain, the collagen-binding domain, the proprotein convertase domain, and the β-jelly roll domain, exhibit collagen-binding or -swelling activity. These activities can induce conformational changes in collagen or the enzyme active sites, thereby enhancing the collagen-degrading efficiency. In addition, thermostable bacterial collagenolytic proteases can function at high temperatures, which increases their degradation efficiency since heat-denatured collagen is more susceptible to proteolysis and minimizes the risk of microbial contamination. To date, only a few thermophile-derived collagenolytic proteases have been characterized. TSS, a thermostable and halotolerant subtilisin-like serine collagenolytic protease, exhibits high collagenolytic activity at 60°C. In this review, we present and summarize the current research on A) the classification and nomenclature of thermostable and mesophilic collagenolytic proteases derived from diverse microorganisms, and B) the functional roles of their C-terminal domains. Furthermore, we analyze the cleavage specificity of the thermostable collagenolytic proteases within each family and comprehensively discuss the thermostable collagenolytic protease TSS.

## Introduction

**Fig. 1:**
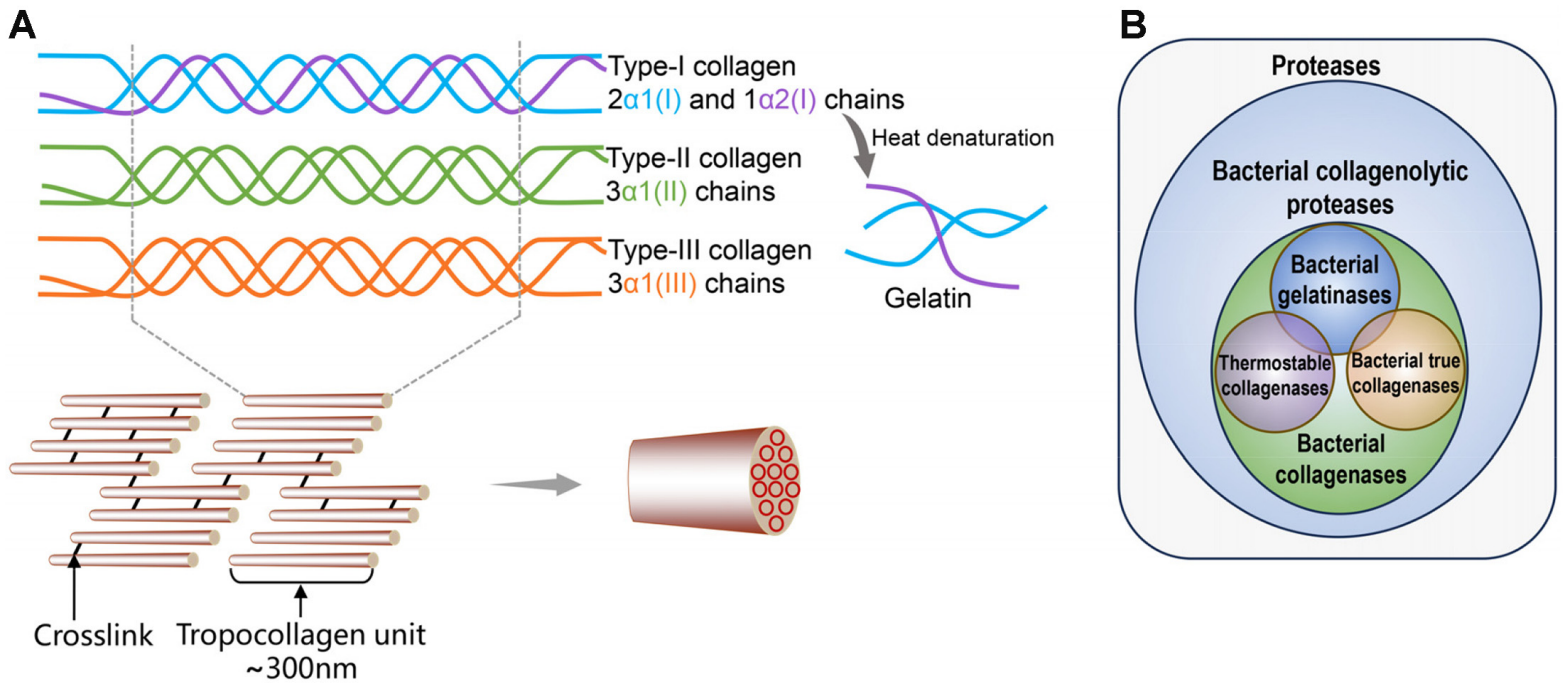
Collagen and its degrading enzymes. (**A**) Structural diagram of different collagens. (**B**) The relationship between collagenolytic proteases, collagenases, true collagenases, and gelatinases in bacteria.

Collagen is a crucial structural protein widely distributed in various extracellular elements, including skin, bone, and dentin, and serving as the major connectivity protein within the vertebrate extracellular matrix (ECM)[1]. Due to its dense structure and water insolubility, which are influenced significantly by stereoelectronic effects and preorganization, collagen resists enzymatic degradation by conventional proteases. Consequently, collagen is susceptible to a limited repertoire of collagenolytic proteases, the cleavage specificity of which is a valuable tool for characterizing and identifying different collagen types [2]. According to published literature, the collagen family consists of 28 distinct members, characterized by a triple-helical architecture comprising three α-chains flanked by nonhelical regions. Collagen includes fibrillar collagen (commonly known as classical fibril-forming collagens, such as types I, II, and III) (Fig. 1A) and nonfibrillar collagen (such as types IV and VI), differentiated by their unique assembly modes of triple helices [3-5]. Generally, fibrillar collagens are assembled from collagen monomers, each comprising three interlaced polypeptide chains to form a right-hand triple helix, whereas nonfibrillar collagens have one or more interruptions in the triple helices [6]. The polypeptide chains in collagen consist of repeating Gly-Xaa-Yaa triplets that stabilize its spiral architecture, with Xaa and Yaa varying; however, they often represent Pro and Hyp, respectively [7]. On the other hand, depending on whether the polypeptide chains in the triple helix are identical, the collagen structure includes both heterotrimeric (*e.g.*, type I collagen, two α1 and one α2 chains) and homotrimeric (*e.g.*, type II collagen, three α1-chains) triple helices, with the heterotrimeric form being more prevalent [8]. At high temperatures, collagen undergoes thermal denaturation, leading to the breakdown of stabilizing bonds in the collagen triple helix and conversion into gelatin [9]. Consequently, it loses its compact texture, making it more susceptible to collagenolysis.

Collagenases have numerous applications in the food, tannery, cosmetic, and meat industries (tenderness is a crucial sensory quality of meat), as well as in the production of pharmaceutical compounds, and even the bio-restoration of frescoes [10]. There is no universally accepted definition for collagenolytic proteases, collagenases, or even gelatinases in bacteria (Fig. 1B). Bacterial collagenolytic proteases are enzymes capable of degrading at least one type of collagen [9]; bacterial collagenases are proteases that cleave the helical region of fibrous collagens under various conditions, such as physiological, thermal or acidic conditions; bacterial true collagenases are proteases that only cleave the helical region of fibrous collagens under physiological conditions (*e.g.*, 37°C and neutral pH), for example, *Clostridium histolyticum* collagenase (ChC) [10]. Heat treatment results in the conversion of collagen to gelatin (the denaturation process of collagen: collagen→monomer→gelatin) (Fig. 1A); therefore, any enzyme degrading collagen at high temperatures is commonly considered to be a gelatinase (gelatinases are produced from a full-length collagenase by the proteolytic removal of C-terminal fragments) [11](Fig. 1B).

Collagenolytic proteases are applied extensively in the food, medical, pharmaceutical, cosmetic, and textile industries [12, 13]. They are commonly derived from animals and bacteria, and can be categorized into three primary groups: mammalian cysteine proteases, bacterial collagenolytic proteases, and mammalian matrix metalloproteinases (MMPs). Cysteine proteases have been discovered in plants, animals, and bacteria, playing roles in various physiological and pathological processes [14]. In animals and humans, they are responsible for senescence and apoptosis, prohormone processing, and animal ECM remodeling [15]. Bacterial collagenolytic proteases comprise four distinct families: bacterial collagenolytic metalloproteinase M9, and bacterial collagenolytic serine proteinase S1, S8, and S53. Notably, bacterial collagenolytic proteases have been predominantly discovered and studied in mesophiles, while those derived from thermophiles are relatively less studied. Psychrophiles and mesophiles flourish under cooler or moderate temperatures, whereas thermophilic microorganisms thrive best at higher temperatures (60–80°C). Hyperthermophiles, on the other hand, prefer extremely hot environments (80–110°C) [16]. Thermostable bacterial collagenolytic proteases from thermophiles or hyperthermophiles have the advantage of effectively degrading collagen and maintaining conformational stability at increased temperatures, thereby decreasing the risk of microbial contamination.

In this review, we have provided brief insights into: (1) the classification and nomenclature of bacterial collagenolytic proteases, including thermostable variants; (2) the functions of their C-terminal domains, as well as the action mechanisms and cleavage specificities of various bacterial collagenolytic proteases on collagen; and (3) a comprehensive understanding of the thermostable subtilisin-like serine protease (TSS) [17], a novel enzyme derived from thermophiles.

## Bacterial Collagenolytic Proteases

**Fig. 2:**
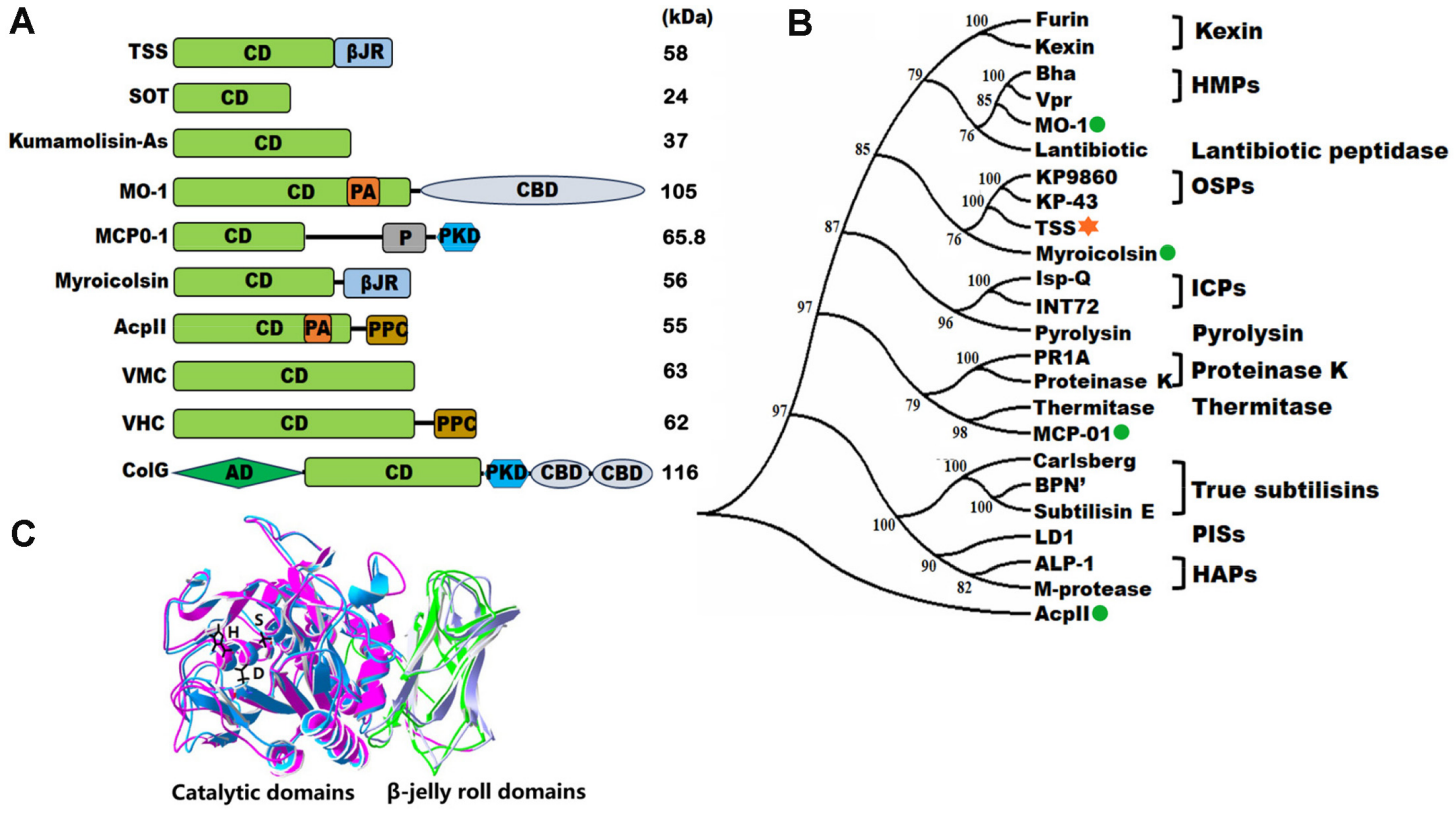
Protein domains and their phylogenetic relationships with typical bacterial collagenases or collagenolytic proteases. (**A**) Schematic representations of the maturase domain organization of typical collagenases or collagenolytic proteases from different microorganisms. All the domain structures were deduced from the amino acid sequences of TSS (OBR56241) from *Brevibacillus* sp. WF146 [17], SOT (BAI44325) from *Streptomyces omiyaensis* [46], kumamolisin-As (BAC41257) from *Alicyclobacillus sendaiensis* [53], MO-1 (AB260948) from *Geobacillus collagenovorans* MO-1 [93], MCP-01 (ABD14413) from *Pseudoalteromonas* sp. SM9913 [67], myroicolsin (AEC33275) from *Myroides profundi* D25 [65], AcpII (AB505451) from *Alkalimonas collagenimarina* [83], VMC (AAC23708) from *Vibrio mimicus* [83], VHC (BAK39964) from *Grimontia (Vibrio) hollisae* [94] and ColG (BAA77453) from *Clostridium histolyticum* [11]. CD: catalytic domain [19]; βJR: β-jelly roll domain [17, 21]; CBD: collagen-binding domain [19]; P: P-proprotein convertase domain [20]; PKD: polycystic kidney disease-like domain [20]; PPC: pre-peptidase C-terminal domain [20]; AD: activator domain [91]; PA: protease-associated domain [92]. (**B**) A rootless phylogenetic tree was constructed from the amino acid sequence alignment of full-length enzymes using the neighbor-joining method in ClustalX and MEGA7 to investigate the evolutionary relationship between TSS and other S8 family subtilases. For the proteases displayed above, the enzymes were divided into the following groups: true subtilisins; HAPs (high-alkaline proteases); ICPs (intercellular proteases); OSPs (oxidatively stable proteases); HMPs (high-molecular-mass proteases); PISs (phylogenetically intermediate subtilisins); thermitase; proteinase K; pyrolysin; and Kexin and lantibiotic peptidase. The origins of the sequences aligned: Kexin (OLN81751) from *Colletotrichum chlorophyti*; furin isoform X1 (XP_011249120) from *Mus musculus* (house mouse); Vpr (M76590) from *Bacillus subtilis*; Bha (G83753) from *Bacillus halodurans* C-125; lantibiotic (KJS88019) from *Desulfosporosinus* sp. BICA1–9; KP-43 (AB051423) from *Bacillus* sp. strain KSM-KP43; KP-9860 (AB046403) from *Bacillus* sp. strain KSM-KP9860; INT72 (P29139) from *Bacillus polymyxa* 72; Isp-Q (Q45621) from *Bacillus* sp. strain NKS-21; pyrolysin (AAB09761) from *Pyrococcus furiosus* DSM 3638; proteinase K (1205229A) from *Parengyodontium album*; PR1A (AAV97788) from *Metarhizium acridum*; thermitase (KAA1806649) from *Bacillus cereus*; subtilisin Carlsberg (2SEC_E) from *Bacillus licheniformis*; BPN’ (Q44684) from *Bacillus amyloliquefaciens*; subtilisin E (P04189) from *Bacillus subtilis* 168; LD1 (AB085752) from *Bacillus* sp. strain KSM-LD1; ALP-1 (Q45523) from *Bacillus* sp. strain NKS-21; M-protease (Q99405) from *Bacillus clausii* KSM-K16; MO-1 (AB260948) from *Geobacillus* sp. MO- 1; myroicolsin (AEC33275) from *Myroides profundi*; MCP-01 (ABD14413) from *Pseudoalteromonas* sp. SM9913; AcpII (AB505451) from *Alkalimonas Collagenimarina*; and TSS (1039472844) from *Brevibacillus* sp. WF146. Collagenolytic proteases from S8 subtilases, including myroicolsin, MCP-01, AcpII, and MO-1, are represented as green dots, whereas TSS is represented using an orange asterisk. (**C**) Homology modeling and structural fitting chart of TSS (RoseTTAFold, https://github.com/RosettaCommons/RoseTTAFold) and KP-43 (PDB code1WMF) by SpdbViewer. The catalytic and βJR domains were represented by light blue and green for TSS, and purple and gray for KP-43, respectively. The side chains of the catalytic triad of TSS (D-H-S) were shown in black.

The bacterial collagenolytic proteases are efficient in cleaving collagen at multiple sites, breaking it down to short peptide fragments. At present, bacterial collagenolytic proteases, mainly including metalloproteinase M9 and the collagenolytic serine proteinase families S1, S8, and S53 [17], are predominantly derived from mesophiles [18]. The major members of each family and their characteristics are summarized in Table 1. The collagen degradation patterns of S1 family collagenases and M9 family *Vibrio* collagenases are somewhat similar to that of MMPs (which function in ECM degradation and cell migration) in the first step of collagen degradation, which is to cleave the peptide bond between Xaa and Gly, lying approximately three-quarters of the way from the N-terminus of collagen. In contrast, other bacterial collagenases, including the M9 family *Clostridium* collagenase and the S8 and S53 family collagenases, possess multiple cleavage sites on collagen , facilitating collagen degradation into oligopeptides and free amino acids [7]. However, the hydrolysis mechanism remains challenging, mainly due to the intricate multi-domain organization of these proteases [11, 17]. Notably, these enzymes comprise a catalytic domain (CD) [19] and, in most cases, at least one C-terminal extension. These C-terminal extensions possess various functional domains, including the collagen-binding domain (CBD) [19], the polycystic kidney disease (PKD)-like domain [20], the pre-peptidase C-terminal (PPC) domain [20], the P-proprotein (P) convertase domain [20], and the β-jelly roll (βJR) domain [17, 21] (Fig. 2A and Table 2).

## M9 Family Collagenolytic Proteases

The M9 family collagenases, primarily derived from *Clostridium* [10, 22-24] and *Vibrio* [25-27], are all zinc-dependent proteolytic metalloenzyme and mainly function at around 37°C. Based on their amino acid sequences and catalytic functions, these collagenases can be divided into two subfamilies: M9A and M9B [28, 29]. The M9A subfamily collagenases comprise class II and III proteases from *Vibrio*. M9B subfamily collagenases include ColA from *Clostridium perfringens* [30], ColT from *Clostridium tetanus* [31], and ColG and ColH from *C. histolyticum*. These metalloproteinases can enzymatically degrade native collagen and play an essential role in degrading the animal ECM, in which cellular–ECM interactions are extremely vital for tissue structure and function [32].

A typical *Clostridium* collagenase comprises an N-terminal activator domain, a catalytic peptidase domain, and several C-terminal recruitment domains [24, 33]. The catalytic peptidase domain contains a conserved zinc-binding motif, while the C-terminal recruitment domains, in most cases, feature one or two CBDs or PKD domains [24, 30].

ChC was the first identified and characterized collagenase, as well as the initial commercially available collagenase for treating adult men with Peyronie’s disease [12, 34]. Additionally, it served as a benchmark enzyme for studies on newly discovered collagenolytic proteases [12, 34]. ChC is a mixture of ColG and ColH. Both fall into the category of true collagenases, exhibiting distinct enzymatic specificities when degrading native collagen [13, 35, 36]. Simultaneously, the CBD of ChC can enhance the efficiency of collagen degradation by facilitating collagen binding [22].

*Vibrio* collagenase, representing the M9A subfamily, has been extensively researched as a crucial virulence factor in certain human bacterial infections. *Vibrio* extracellular proteases consist of three distinct classes, each characterized by its collagen and casein degradation patterns. Class I proteases exhibit the highest activity toward elastin yet cannot digest either collagen or casein, belonging to the M4 family (thermolysin) [26, 29]. Class II (VMC) and III (VHC) (Fig. 2) proteases are collagenases and can degrade native collagen, yet they exhibit notable differences in function and structure [7]. Class II collagenase possesses a zinc-binding motif (HEYTH), but lacks a C-terminal domain [37, 38], whereas the FAXWXXT motif in the carboxyl terminus of *Vibrio mimicus* collagenase in this class is associated with collagen binding [27]. On the other hand, class III collagenase possesses a HEYVH motif and contains the PKD and PPC domains [25, 26, 39]. Notably, neither the PPC nor PKD domain of class III collagenase exhibits collagen-binding activity, and their actual functions deserve further study.

ColG and ColH act on the distinct hyperreactive sites Yaa-Gly within the repetitive Gly-Xaa-Yaa collagen sequence [40], while *Vibrio* collagenases target a point that is three-quarters of the way from the N-terminus of collagen by cleaving the peptide bond of Gly-Xaa [41]. In summary, the collagen-binding mechanisms of *Clostridial* and *Vibrio* collagenases may be different, warranting further investigation [12].

Recently, the crystal structure of *Grimontia hollisae* collagenase (Ghcol) complexed with its substrate (Gly-Pro-Hyp-Gly-Pro-Hyp, GPOGPO) was determined in an attempt to understand the catalytic mechanism. Combining active-site geometry and site-directed mutagenesis, this study revealed that Glu493 and Tyr564 were essential for catalysis [42]. Moreover, a structure-based report revealed the collagenase module of *Vibrio* collagenase VhaC (an M9A collagenase with optimal enzymatic reaction conditions at 40°C and pH 8.0), recognizing the triple-helical collagen by its activator domain, followed by the subsequent cleavage by the peptidase domain along with the closing movement of the collagenase module. This mechanism is different from the proposed collagenolysis of *Clostridium* collagenase [43]. Another new M9A collagenase, VP397, from marine *Vibrio pomeroyi* strain 12613, can hydrolyze various collagenous substrates, including fish collagen, mammalian collagens of types I to V, etc., with the highest activity at 40°C and pH 8.0. Results also showed that VP397 first assaults the C-telopeptide region to dismantle the compact structure of collagen and dissociate tropocollagen fragments, which are further digested into peptides and amino acids by VP397, mainly at the Yaa-Gly bonds in the repeating Gly-Xaa-Yaa triplets. In addition, domain deletion mutagenesis showed that the catalytic module of VP397 alone is capable of hydrolyzing type I collagen fibers and that its C-terminal PPC2 domain functions as a CBD during collagenolysis [19].

Additionally, a collagenase was found in *Lysinibacillus sphaericus* VN3 and characterized by its collagenase activity, which was significantly enhanced by Zn^2+^. The optimum temperature and pH for this collagenase were approximately 37°C and pH 7.0, respectively, but activity was lost between 50°C and 60°C [44]. In addition, a heat-resistant (up to 100°C) Zn-metalloprotease with collagenase activity was identified and characterized from *Mannheimia haemolytica* A2 [45]. Both of these enzymes have a molecular mass of approximately 110 kDa and are highly likely members of the M9 family of collagenolytic proteases.

## S1 Family Collagenolytic Proteases

Literature on S1 family bacterial collagenolytic proteases (chymotrypsin) is scarce. A distinct feature of this family is the presence of the protease catalytic triad His-Asp-Ser (H-D-S). Notably, some members in this family, namely serine protease from *Streptomyces omiyaensis* (SOT) and *Streptomyces griseus* trypsin (SGT), are categorized as true collagenases. The structure of SOT comprises a catalytic domain without C-terminal domains (Fig. 2). Uesugi *et al*. [46] discovered that the N-terminal domains of SOT and SGT are linked to their specificity toward structural protein substrates. SOT can hydrolyze both type I and type IV collagens with marked efficiency at 37°C; however, SGT exhibits higher hydrolytic activity toward type I collagen rather than type IV collagen.

## S53 Family Collagenolytic Proteases

The S53 (sedolisin) family collagenolytic proteases have been identified in various organisms and are characterized by significant activity under conditions of high temperature and low pH, rather than in the neutral to alkaline region where subtilisin is active [47, 48]. A distinct feature of this family is the protease catalytic triad Ser-Glu-Asp (S-E-D) [49]. This feature distinguishes it from the prototypical catalytic triad Asp-His-Ser (D-H-S) found in subtilases (S8 family) [50]. Initially isolated from thermoacidophilic soil bacterium *Alicyclobacillus sendaiensis* NTAP-1, kumamolisin-As is a thermostable protease within the S53 family that exhibits maximum collagenolytic activity under conditions of pH 4.0 and 60°C [51-53]. Similar to SOT in the S1 family, the architecture of kumamolisin-As includes a catalytic domain while lacking a CBD. Kumamolisin-As contains an unusual substrate-binding pocket, exhibiting a preference for degrading collagen with a loose structure under conditions of high temperature and low pH [54]. It also demonstrates a strong preference for Arg at the P_1_ site on collagen due to a negatively charged residue (Asp179) in the substrate-binding pocket [55]. Furthermore, kumamolisin-As exhibits remarkable thermostability and acid resistance, retaining over 80% of its initial activity even after incubation for 1 h at pH 4.0 and 60°C [53]. Notably, kumamolisin-As primarily degrades denatured collagen at 60°C (type I collagen, for instance, is thermally unstable at that temperature or even at 37°C) [56]. Another S53 family collagenolytic protease, kumamolisin-Ac, was purified from thermoacidophilic bacterium *Alicyclobacillus acidocaldarius*. It can efficiently hydrolyze type I collagen at pH3.0 and 60°C [57].

Due to their activity under acidic pH and high-temperature conditions, which can effectively eliminate microbial contamination of the reaction system, and a BLAST search of the kumamolisin-As amino acid sequence revealing a set of S53 family proteins, further investigation is required to confirm whether these proteins are bacterial collagenolytic proteases.

## S8 Family Collagenolytic Proteases

There are a total of 14 clans of serine proteases based on the amino acid sequences, tertiary structures, and the order of the catalytic residues. Family S8 belongs to clan SB, which possesses the catalytic residue known as the Asp-His-Ser (D-H-S) triad. In family S1, the catalytic triad is composed of His-Asp-Ser (H-D-S), while family S53 features the catalytic triad Ser-Glu-Asp (S-E-D) [50].

MO-1, derived from the thermophilic bacterium *Geobacillus collagenovorans* MO-1, stands out as the first identified collagenolytic protease within the S8 family (the subtilisin or subtilase family) [58, 59]. Displaying exceptional thermostability, MO-1 efficiently functions over a wide temperature range (25–80°C), with an optimum temperature of 60°C [58]. This protease exhibits robust activity against type I and type IV collagens, effectively breaking them down into various small fragments, implying its involvement in collagen degradation at multiple sites [58]. In addition to its catalytic domain, MO-1 also features a CBD (Fig. 2A). Consequently, it can bind to collagen but not to other insoluble substrates, such as elastin or keratin [59]. Furthermore, a recently discovered alkaline protease, AcpII, derived from the deep-sea bacterium *Alkalimonas collagenimarina* AC40^T^, demonstrates optimal collagen digestion at 45°C under alkaline conditions (pH 8.5–9.0) [60]. AcpII contains a protease-associated (PA) domain, involved in protein-protein interaction, which does not directly bind to collagen but coordinates substrates to the active site, thereby enhancing the collagen affinity of the catalytic domain to some extent [60]. Meanwhile, a thermostable serine collagenolytic protease derived from *Thermoactinomyces* sp.21E can effectively digest type I collagen at 60°C; however, it exhibits no activity toward elastin [61].

MCP-01 from *Pseudoalteromonas* sp. SM9913 and myroicolsin from *Myroides profundi* D25, both isolated from deep-sea bacteria, exhibit optimal collagenolytic activity at 60°C and pH 8.5–9.0 [62-65]. MCP-01, with a catalytic domain capable of degrading collagen by itself, albeit less efficiently than the intact protease, contains proprotein (P) convertase and PKD domains in its C-terminus (Fig. 2A) [64, 66]. In fact, the PKD domain of MCP-01 can bind and swell collagen, enhancing the degradation efficiency of the catalytic domain on collagen [63, 67]. On the other hand, myroicolsin contains the βJR domain in its C-terminus. Interestingly, this domain does not exhibit collagen-binding activity, suggesting that myroicolsin may not rely on this domain for collagenolysis [65]. The mechanisms of myroicolsin and MCP-01 are comparable in terms of collagen degradation [65, 67]. In addition to type I collagen, MCP-01 and myroicolsin can both degrade type II and type IV collagens, fish scale collagen, and gelatin [62-65]. Nevertheless, when acting on native and denatured collagen, they exhibit distinct degradation properties [65, 67]. In general, myroicolsin cleaves the Gly-Xaa peptide bond on native collagen, while it often targets peptide bonds with Lys or Arg in the P_1_ position on denatured collagen, with the P1’ position consistently comprising Gly [65]. On the other hand, MCP-01 exhibits a non-strict preference for peptide bonds characterized by the presence of Pro or basic residues at the P_1_ site and/or Gly at the P1’ site on collagen [64]. Both share a preference for cleaving the Gly-Xaa peptide bond, which is abundant in collagen, explaining their remarkable collagenolytic activity [65, 68]. Importantly, MO-1, MCP-01, and myroicolsin can degrade native type I collagen at temperatures at or below 37°C. Therefore, all can be considered true collagenases.

In addition, an extracellular subtilisin-like serine protease named SPSFQ (belonging to the S8 family of collagenolytic proteases) from *Acinetobacter baumannii* was isolated from fermented food [69]. Recombinant expression and characterization revealed that SPSFQ catalyzes casein at an optimum temperature of 40°C (varying from 20 to 70°C) and pH 9.0. Its activity is stimulated in the presence of Ca^2+^ and severely inhibited by PMSF. SPSFQ is capable of degrading several tissue-associated protein substrates, exhibiting the highest catalytic activity for casein, moderate activity for gelatin and azure keratin, and low activity for fibrin and azocoll.

## The Thermostable Subtilisin-like Serine Protease TSS

Numerous microbial collagenases have been discovered, primarily in mesophiles. However, due to their poor thermostability, only a few known bacterial collagenases have practical applications [53]. Therefore, it is scientifically and practically important, for research and industry, to explore thermostable and/or thermophilic collagenolytic proteases and study their resistance to high temperatures as well as their underlying action mechanisms on collagen. Thermostable collagenolytic proteases exhibit remarkable enzymatic stability at high temperatures, efficiently degrading different types of collagens and collagen-like substrates. Understanding the different mechanisms by which thermophilic and mesophilic collagenolytic proteases degrade collagen is vital for comprehending thermal adaptation mechanisms and degradation patterns in thermophiles. However, to date, only a small number of thermophilic collagenolytic proteases have been comprehensively investigated (Table 1).

**Fig. 3:**
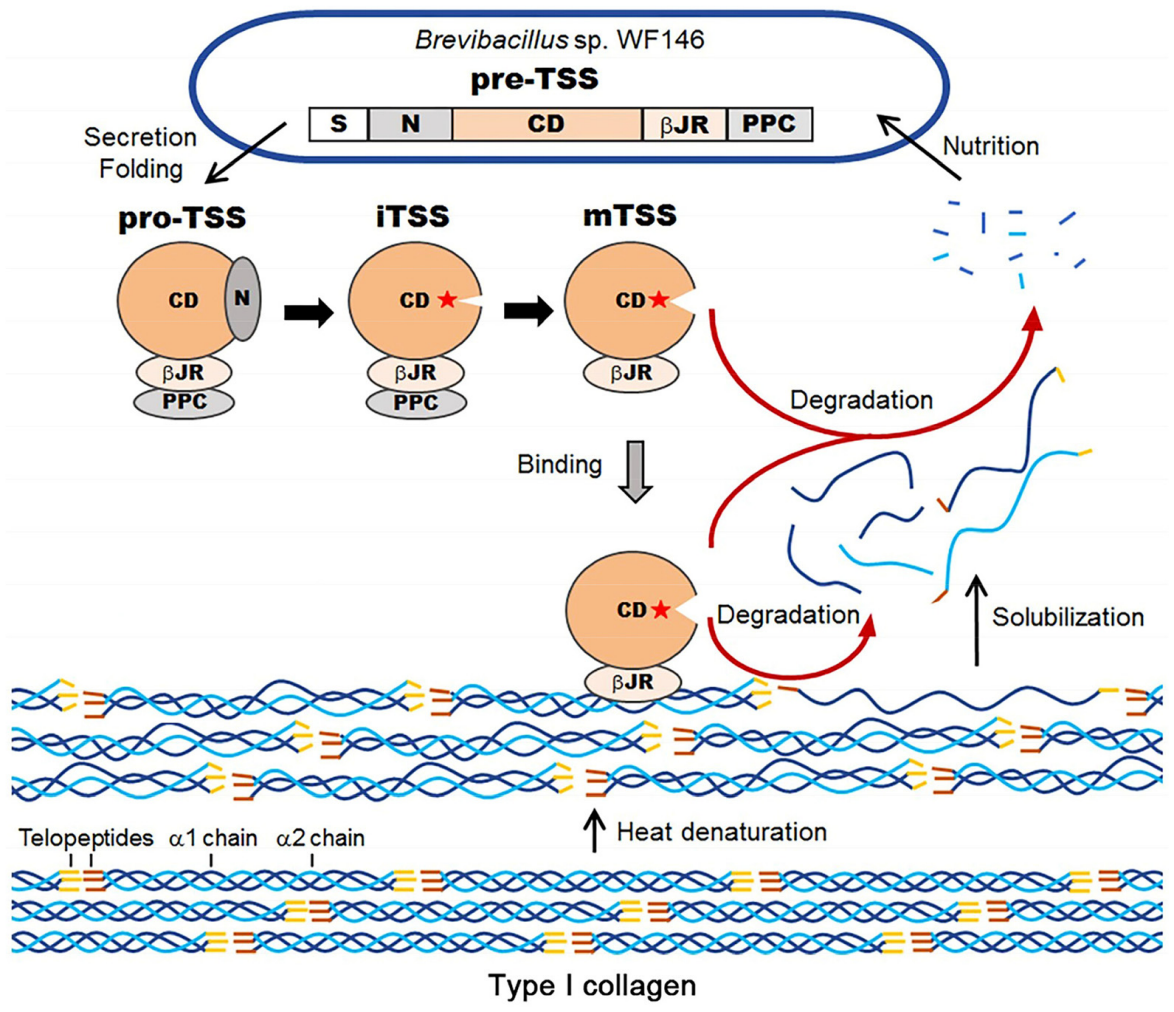
Schematic diagram of the autoprocessing maturation of TSS and its degradation on type I collagen at high temperatures. TSS is synthesized as a precursor (pre-TSS) within *Brevibacillus* sp. WF146 and then secreted outside the host by a signal peptide. After being cleaved by a specific signal peptidase, the precursor folds into a proprotein (pro-TSS), which subsequently converts into an intermediate (iTSS) by removing the N-terminal propeptide. Finally, it becomes a mature enzyme (mTSS) by removing the PPC domain. At room temperature, collagens exist in a naturally triple-helical state. However, under high-temperature conditions, their molecular structure becomes loosely packed, resulting in the dissolution of some collagens into the solution. In solution, mTSS can bind to the surface of soluble collagens and degrade them. Additionally, when mTSS is adsorbed onto insoluble substrates, it degrades both insoluble and soluble collagen. The small degradation products can be absorbed and utilized by the host for metabolism.

TSS is a thermostable and halotolerant subtilisin-like protease derived from the thermophile *Brevibacillus* sp. WF146. It can withstand extreme conditions and has emerged as one of the most thermostable and halotolerant collagenolytic proteases reported to date (Fig. 3) [17, 70]. Notably, TSS demonstrates thermostability comparable to that of kumamolisin-As, retaining approximately 80% of its initial activity even after a 30-min incubation at 75°C [17]. TSS exhibits optimal activity at 70°C and pH 9.0, with a half-life of 1.5 h at 75°C. Furthermore, TSS exhibits halotolerance to NaCl concentration up to 4 M; this property is similar to that of haloarchaeal proteins and the newly discovered halotolerant protease, Als [70].

The precursor of this multidomain protease comprises a signal peptide, an N-terminal peptide, a subtilisin-like catalytic domain, a βJR domain, and a PPC domain [17]. The PPC domain is not vital for cleaving the N-terminal propeptide, while the βJR domain contributes to TSS folding, stability, and activity. Unlike the PKD domain of MCP-01 [63] and the PPC domains of several serine proteases and metalloproteases from psychrotolerant/mesophilic bacteria [71], which can bind and swell insoluble collagen, the PPC and βJR domains of TSS can bind but not swell insoluble collagen. The collagen-swelling function of the PPC or βJR domain seems unnecessary for TSS, as collagen unwinds under high temperatures [17, 72]. Although the PPC domains in collagenolytic proteases can expose collagen, they do not disrupt its crosslinks or unwind the collagen triple helix [63]. βJR is essential for cleaving the N-terminal pro-domain of KP-43 [21]. It participates in the folding and stability of TSS, as well as in collagen binding and collagenolytic activity [17]. Notably, βJR with its Ca sites is required for the hyperthermostability and halotolerance of the protease [17, 73].

As reported, PKD or PPC domains can enhance catalytic efficiency of collagenases and can be utilized as biological swelling agents in food processing, indicating wide-ranging application prospects in medicine, pharmacy, cosmetics, and food industry in the future [20]. Based on the thermostability and halotolerance of collagenolytic proteases like TSS with its βJR domain, they should be further explored for their potential as innovative, therapeutic agents in modern biomedicine for use in wound healing, fibrotic and scarring processes, collagen-induced arthritis, and other diseases [74, 75]. Specifically, such collagenolytic proteases could be useful, considering that the intracellular environment is crowded with macromolecules (high concentrations of background molecules) [76], and that the temperature of the affected tissue is usually 1–1.5 degrees higher in inflamed tissue than in normal tissue [74]. Additionally, thermostable bacterial collagenolytic proteases could be potential candidates for multifaceted applications such as those in food and meat industries, fish scales, or processing leather at high temperatures, etc. This has the advantages of increasing degradation efficiency because heat-denatured collagen is more susceptible to proteolysis and minimizes the risk of microbial contamination.

TSS belongs to the S8 family proteases, as supported by the following evidence: (1) TSS possesses the typical catalytic triad Asp-His-Ser (D-H-S); (2) TSS is closely related to KP-43, an oxidation-resistant protease among the general subtilisin-like proteases [17], in the rootless phylogenetic tree of subtilases (Fig. 2B). (3) TSS and KP-43 share high sequence identity (49% for the catalytic domain and 44% for the βJR domain) [17], and they are much similar in spatial structure (Fig. 2C). Furthermore, TSS is a unique collagenolytic protease for the following reasons: (1) TSS exhibits little activity toward azocoll or type I collagen at 37°C, but shows increased collagenolytic activity with rising temperature up to 70°C. TSS has a strong preference for Arg in the P_1_ position and Gly in the P1’ position, particularly on insoluble rather than thermally solubilized heat-denatured collagens (Fig. 3) [17]; (2) TSS possesses a high acidic amino acid residue content (16.4%), contributing to its increased thermostability and halotolerance. In summary, TSS is a thermostable and halotolerant subtilisin-like collagenolytic protease, which prefers to degrade insoluble heat-denatured collagens at elevated temperatures.

## Conclusion and Prospects

Collagen and collagen peptides are valuable biomaterials owing to their diverse biochemical and medical functions, commercial utility, and involvement in some human diseases [75, 77]. Collagenolytic proteases, which are capable of hydrolyzing native or denatured collagen, have been identified in various organisms, including mammals, microorganisms, plants, fungi, vertebrates, and the larvae of worms and crabs. Bacterial collagenolytic proteases can cleave collagen at multiple sites, yielding different types of degradation products. The application of mesophilic collagenolytic proteases is limited, partly because they are prone to association with pathogenic bacteria that invade mammalian cells. In these cells, type I collagen constitutes approximately 95% of the structural molecules in many animal tissues [78]. In contrast, thermophilic collagenolytic proteases are rarely related to pathogenesis [79], making them ideal model enzymes for exploring the action mechanisms of mesophilic collagenolytic proteases in human diseases. Therefore, most research findings have focused on elucidating bacterial collagenolytic proteases, primarily those derived from thermophiles. Thermostable collagenolytic proteases also possess scientific and practical importance in several industries, including leather production, food processing, cosmetics, and pharmaceuticals [12]. Moreover, some bacterial collagenolytic proteases function as crucial virulence factors in human diseases due to their ability to digest collagen in the ECM, and their emergence as attractive targets for overcoming antimicrobial resistance has recently garnered further attention [80].

Owing to the ability of thermophilic bacterial collagenolytic proteases like TSS to withstand several extreme conditions, including high temperatures, increased salinity, and alkaline environments, gaining insight into the isolation and elucidation of their properties holds promising applications in fish waste disposal and the leather industry [81]. Further investigations can focus on the following aspects: (1) The primary challenge is how to obtain a thermophilic bacterium containing thermophilic collagenase. Thermophile *Brevibacillus* sp. WF146 was isolated from the soil of a campus with a subtropical monsoon climate where the highest temperature exceeds 40°C in summer. Such locations can be alternative sources for discovering more novel thermostable collagenolytic proteases under extreme conditions. (2) Systems biology-based analysis can provide a powerful platform for identifying potential thermostable collagenolytic protease-encoding genes. However, it is worth noting that some domains were not always necessary for collagen-binding or -swelling, as shown in previous studies. For instance, the PPC and βJR domains of TSS can bind but not swell collagen; the βJR domain of myroicolsin cannot bind collagen; the FAXWXXT motif in the CD of *V. mimicus* collagenase is associated with collagen binding, even though the protease has no C-terminal domain at all. (3) Most methods used to understand the heat resistance mechanisms of thermostable bacterial collagenolytic proteases were biochemistry-based. To select new appropriate methods based on structure characteristics, crystal diffraction, directed evolution, gene knockout, and gene modification, collaboration among researchers from different disciplines is needed. Such collaboration is the best way to clarify the adaptation strategies of thermophiles and improve the thermostability of their engineered enzyme variants, thereby boosting collagen degradation efficiency and extending the half-lives of these enzymes. (4) Finally, developing the production, environment, and practical applications of these newly identified bacterial thermostable collagenolytic proteases based on unique requirements and conditions would be of great interest to research and industry.

## Figures and Tables

**Table 1:** Characteristics of representative collagenolytic and thermostable collagenolytic proteases from diverse microorganisms.

Collagenolytic proteases	Optimal temperature (°C) and pH	Size (kDa)	Family
TSS from *Brevibacillus* sp. WF146 [17]	70°C; pH 9.0	58.0	S8
MO-1 from *Geobacillus collagenovorans* MO-1 [82]	60°C; pH 7.1-9.3	105.0	S8
MCP-01 from *Pseudoalteromonas* sp. SM9913 [67]	60°C; pH 9.0	65.8 [66]	S8
AcpII from *Alkalimonas collagenimarina* AC40^T^ [83]	45°C; pH 8.5-9.0	55.0	S8
21E from *Thermoactinomyces* sp.21E [84]	70-75°C; pH 9.0-9.5	50.0	S8
Myroicolsin from *Myroides profundi* D25 [65]	60°C; pH 8.5	56.0	S8
SPSFQ from *Acinetobacter baumannii* [69]	40°C; pH 9.0	30.0	S8
SOT from *Streptomyces omiyaensis* [46]	55°C; pH 8.0-9.0	24.0	S1
SGT from *Streptomyces griseus* ATCC 10137 [46]	55°C; 7.5-8.6	24.0	S1
Kumamolisin-As from *Alicyclobacillus sendaiensis* NTAP-1 [53]	60°C; pH 4.0	37.0	S53
Kumamolisin-Ac from *Alicyclobacillus acidocaldarius* [85]	60°C; pH 2.0	45.0	S53
ColG/ColH from *Clostridium histolyticum* [11]	37°C; pH 7.4 (6.3-8.0) [74]	116.0	M9B
ColT from *Clostridium tetani* E88 [86]	37°C; pH 7.4 (6.3-8.0) [74]	114.0 [31]	M9B
ColA from *Clostridium perfringens* [30]	42°C; pH 7.0-7.2	116.0	M9B
VMC from *Vibrio mimicus* [87]	30-40°C; pH 8.0 [88]	63.0	M9A
VHC from *Grimontia (Vibrio) hollisae* [89]	30-40°C; pH 7.0-8.0	62.0	M9A
MPK from *Bacillus subtilis* strain MPK [90]	60°C; pH 7.5	61.0	Cysteine protease

**Table 2:** The categories and functions of the domains in typical collagenolytic proteases.

Domain	Function
CD	It is responsible for the enzymatic activity of the collagenase and contains the active site where the cleavage of collagen molecules occurs [19].
CBD	It facilitates the binding of the enzyme to collagen substrates, enhancing the efficiency of collagen degradation by bringing the enzyme into close proximity to its target [19].
PKD/PPC	PKD and PPC domains belong to Immunoglobulin-like (Ig-like) beta-sandwich protein. These domains can swell insoluble collagen and release collagen fiber, which is more easily degraded by collagenases [20].
AD	It mediates the initial recognition of soluble collagen and unwinds collagen locally, transiently, and reversibly [91].
PA	It has collagen-binding ability [92].
βJR	It is referred to as the 'P-domain' and is essential for cleaving the N-terminal pro-domain [21]. It participates in enzyme folding and stability, collagen binding, and collagenolytic activity [17]. It is required for the hyperthermostability of protease [17, 73].
P	P-proprotein convertase domain is necessary to keep the protease structure stable [20].
